# Propolis and Its Potential to Treat Gastrointestinal Disorders

**DOI:** 10.1155/2018/2035820

**Published:** 2018-03-15

**Authors:** Luisa Mota da Silva, Priscila de Souza, Soad K. Al Jaouni, Steve Harakeh, Shahram Golbabapour, Sérgio Faloni de Andrade

**Affiliations:** ^1^Núcleo de Investigações Químico-Farmacêuticas (NIQFAR), Universidade do Vale do Itajaí (UNIVALI), Rua Uruguai 458, Centro, 88302-202 Itajaí, SC, Brazil; ^2^Department of Pediatric Hematology/Oncology, Faculty of Medicine, King Abdulaziz University Hospital (KAUH), King Abdulaziz University (KAU), Jeddah, Saudi Arabia; ^3^Yousef Abdullatif Jameel Research Chair for Prophetic Medicine Application, Faculty of Medicine, KAUH, KAU, Jeddah, Saudi Arabia; ^4^Special Infectious Agents Unit, King Fahd Medical Research Center, King Abdulaziz University, Jeddah, Saudi Arabia; ^5^Shiraz Transplant Research Centre, Shiraz University of Medical Sciences, Shiraz, Iran

## Abstract

There are a number of disorders that affect the gastrointestinal tract. Such disorders have become a global emerging disease with a high incidence and prevalence rates worldwide. Inflammatory and ulcerative processes of the stomach or intestines, such as gastritis, ulcers, colitis, and mucositis, afflict a significant proportion of people throughout the world. The role of herbal-derived medicines has been extensively explored in order to develop new effective and safe strategies to improve the available gastrointestinal therapies that are currently used in the clinical practice. Studies on the efficacy of propolis (a unique resinous aromatic substance produced by honeybees from different types of species of plants) are promising and propolis has been effective in the treatment of several pathological conditions. This review, therefore, summarizes and critiques the contents of some relevant published scientific papers (including those related to clinical trials) in order to demonstrate the therapeutic value of propolis and its active compounds in the treatment and prevention of gastrointestinal diseases.

## 1. Introduction

Propolis or “bee glue” is a resinous waxy-like substance. Honey bees produce it by mixing their saliva and beeswax with the exudates obtained from plants like tree buds, sap flows, leaves, branches, and barks of plants found in the vicinity of the beehive. The ultimate goal of propolis is for bees to protect their hives by utilizing it to seal cracks and protect bees from predators and microorganisms and provide thermal insulation [1–3]. The term propolis has originated from Greek word pro, for or in defense of, and polis, the city [1]. The color of propolis is variable and depends on the plants' type that the bees used to collect the resinous substances. Three main colors have been noted: green, red, brown, or black propolis [4]. For instance, the red propolis from Cuba or Venezuela has botanical origins identified as* Clusia nemorosa* Forsteronia G. Mey (Clusiaceae) and* Clusia scrobiculata* Benoist (Clusiaceae), respectively. Red propolis from Northeastern Brazil has* Dalbergia ecastaphyllum* (L.) Taub. (Leguminosae) as botanical source while Brazilian green propolis originates mainly from* Baccharis dracunculifolia* DC (Asteraceae). Therefore, the geographical location, plant sources, collection season, bee species, and solvents used in the extraction have an influence on the chemical composition and on the pharmacological activity of propolis preparation. Despite this wide range of its composition, the records indicate that propolis has been used in the folk medicine since 300 BC [5]. In the last decades, it has attracted the interest of researchers around the world because of its several biological and pharmacological properties, with over 2500 articles being published on Pubmed website (https://www.ncbi.nlm.nih.gov/pubmed/) about this substance over the last 30 years. Moreover, it has gained popularity as either an alternative medicine or as a dietary supplement for health amelioration and disease prevention in various parts of the world, including the United States of America, European Union, Brazil, and Japan [6]. Nowadays, propolis has been widely used to treat several illnesses including those that affect the gastrointestinal tract, such as mucositis, colitis, gastritis, and peptic ulcer [7–10]. This is in addition to its potential to treat different forms of gastrointestinal cancer, as presented in this article. Thus, the aim of this review is to summarize and critique published articles related to studies on the use of propolis and its main active ingredients in the treatment of gastrointestinal disorders and other related disorders.

## 2. Methodology

Considering the main gastrointestinal disorders that propolis is normally used to treat, a search has been conducted on Pubmed (https://www.ncbi.nlm.nih.gov/pubmed/), Science Direct (http://www.sciencedirect.com/), and Medline (https://www.nlm.nih.gov/bsd/pmresources.html) databases using the terms “propolis and ulcer, propolis and gastroprotective, propolis and mucositis, propolis and colitis, propolis and gastrointestinal cancer.” Relevant articles have been included in this review.

## 3. Propolis in the Treatment of Oral Mucositis

Oral mucositis (OM) is an inflammation of the oral mucosa of the mouth. OM is observed in cancer patients, especially those with squamous cell carcinoma located in the head and neck area, when treated by chemo and/or radiotherapy [11–14]. OM is one of the most serious complications that are facing cancer patients [15]. Many possible age and gender related complications result from OM. It has been reported that older patients have less ability to repair the damaged DNA associated with treatment and are, thus, more at risk of developing problems. On the other hand, younger patients have the ability to deal better with OM because they have a faster rate of proliferation of the epithelial cells and this will be an important factor in dealing with OM [16]. Females are more at risk of developing OM than males. Various risk factors have been reported including cigarette smoking, excessive alcohol intake, defective restorations, orthodontic appliances, ill-fitting prostheses, and other mucosal irritations [17]. The related risk factors are associated with the area of oral mucosal treated and the type, dose, and intensity of the chemotherapy used [18]. This is in addition to the frequent daily and repetitive radiation treatment [16].

The aggressive medical agents, such as cisplatin and 5-fluorouracil (5-FU), when used in the presence or absence of radiation therapy result in the development of OM in comparison to the use of “gentler” agents like gemcitabine [11]. The OM induced chemotherapy usually manifests itself during the first week after the beginning of therapy and peaks in the 2nd week. It appears first by thinning of oral tissues that leads to erythema. As these tissues become thinner, ulceration eventually occurs [14]. Potential complications include pain, increased risk of local and systemic infections, bleeding, and insufficient food intake, which may lead to breaks in treatment sessions [15].

The typical manifestations associated with OM include the following: atrophy, erythema, ulceration, and swelling of the mucosa [19]. Such manifestations are accompanied with pain, elevated risk of infection, and dysphasia and may lead to dehydration and malnutrition [13, 16, 17, 20]. Other medically related problems include xerostomia and sensational changes that may lead to reduced food intake and finally result in anorexia, malnutrition, and body mass loss and weakness [13].

The traditional way to manage OM is to educate the involved patient to comply with treatment and give the patient good nutritional support, hydration, use of saline rinses, topical and systemic pain relief, and infection surveillance [17]. So far, no therapy has been effective against OM. However, infections associated with OM are usually treated with antibiotics and antifungal agents. The short-term use of antibiotics will lead to the stabilization of resistant bacteria in the human gut for many years and may cause lots of complications associated with treatment [21, 22].

In this section, the role of the external use propolis in the treatment of OM and related mouth diseases will be discussed. The external use of propolis is defined by the application of pharmaceutical or natural products on the surface or point of illness [23]. External uses of propolis (EUP) include the use of pharmaceutical, cosmetic, and oral products such as ointment [24], gel [25], and mouthwash [26].

A recently published systematic review on propolis for oral health reported that it can reduce oral infection and dental plaque and treat stomatitis [27]. In another study, which evaluated the efficacy of ethanolic extract of propolis on radiation-induced mucositis in rats, propolis was found to effectively reduce and/or delay radiation-induced mucositis in an animal model. However, it is recommended that further studies need to be conducted to further confirm this effect [28].

Not all of the published studies have reported the geographical locations from where the propolis was collected [29–36]. In only one study the chemical composition of propolis has been mentioned which gives this study importance since it characterized the propolis tested and listed its chemical composition [30]. It has been reported that all the propolis from different areas had similar composition but its efficacy was concentration dependent [37]. In addition, different components were identified in propolis collected from different regions from the same country [38] and their adverse effects were identified from certain countries [39]. Based on that, the geographical location is a key factor in the safety and efficacy of propolis [40].

In studies where placebo was used, they used the same form in the control group without hinting to anything about taking into account the smell of the propolis [29, 30, 36, 41]. It would have been prudent to utilize indistinguishable placebo as compared to the experimental treatment. Considering the scent is crucial, since propolis has a distinct aromatic smell and subjects are usually familiar with its distinctive smell; this characteristic should be taken into consideration in future blind studies on propolis [42].

Propolis when used as an ingredient in mouthwashes showed protection against oral disease which is likely due to its antimicrobial efficacy [43]. There was no significant difference in the efficacy that is provided by propolis when used as a gel or as a mouthwash [44] or as a buccal paste [45].

We have published an open labeled randomized controlled recent study on the use of Saudi honey that is similar in so many ways to propolis on 40 pediatric cancer patients undergoing chemo/radiotherapy. The topical application of local Saudi honey resulted in a significant reduction of OM, associated with bacterial and fungal (candida) infections. The use of honey in the treatment of the patients has led to a reduction in the hospitalization time accompanied by a significant gain in body weight, delayed onset, reduced infections, and decreased severity of pain related to OM [46].

## 4. Propolis in the Therapeutic Management of Ulcerative Colitis

Ulcerative colitis (UC), a subtype of inflammatory bowel disease, is a chronic inflammatory condition that causes a constant inflammation of the colonic mucosa. It is characterized by significant morbidity and worsening in the quality of life of the affected patients [47, 48]. Although the exact etiology is unidentified, several authors suggest that an interaction between genetic and environmental factors, as well as a dysregulated immune system, can result in mucosal inflammation [49, 50]. The main clinical symptoms of UC are abdominal pain, diarrhea, and rectal bleeding, which are currently treated with mesalamine (5-aminosalicylic acid or 5-ASA), corticosteroids, immunosuppressants, antibiotics, and biologic therapies [such as the antitumor necrosis factor (TNF) agents]. However, the effectiveness of the available medical therapy and the list of a large number of important side effects are the two major concerns in clinical practice for the efficient and safe management of UC [48, 51–53]. Studies with novel therapies based on medicinal plants have been the focus of a pronounced number of investigations in the last years, which points out promising results in experimental trials (for review see [54]). In this sense, propolis and its active compounds have already been the target of several preclinical studies about its advantage in the treatment of UC.

The first evidence about the beneficial role of propolis on experimental UC was described in 1979 [55]. Since then, other studies have been conducted either with propolis or with its active components, in different animal models with induced UC. In 2007, Aslan et al. [56] showed the effectiveness of propolis in a model where acetic acid was used to induce colitis in rats. The intracolonic instillation of acetic acid is the simplest and most reproducible model of many characteristics of the human colitis [57]. In that study, propolis treatment was effective in attenuating UC, by mechanisms associated with decrease in oxidative stress and inflammation, which are key parameters in the pathogenesis of the disease [56]. Subsequently, the same group of researchers explored the effect of propolis on bacterial translocation using the same model of acetic acid induced experimental colitis. There are several evidences showing that luminal bacteria are involved in mucosal inflammatory responses in UC, whereby this bacteria causes the disruption of the intestinal mucosa barrier integrity [58]. Thus, the authors concluded that propolis was able to reduce bacterial translocation, due to its ability to restrict the damage caused by acetic acid induction, and results in the protection of the integrity of the intestinal wall [59]. More recently, another study using the same model of acetic acid-induced colitis in rats showed that the hydroalcoholic extract of Brazilian red propolis attenuated colitis, an effect associated with decreases in myeloperoxidase (MPO) activity, gross, and histological scores of tissue damage and the inducible isoform of nitric oxide synthase (iNOS) expression [60].

The properties of propolis were also evaluated in other experimental models, including the trinitrobenzene sulfonic acid- (TNBS-) induced colitis. TNBS intrarectal inoculation is capable of activating the immune response driven intestinal inflammation, which is characterized by infiltration of the lamina propria with CD4+ T cells, neutrophils, and macrophages [61–63]. Okamoto et al. showed the suppressive effect of Brazilian propolis on Th1 differentiation, an action related to a reduction on the severity of TNBS-induced UC in mice [64]. Additionally, the effects of propolis hydroalcoholic extract were also explored using TNBS-induced UC in rats, in which the decreases in the inflammatory infiltrate and the number of cysts and abscesses in the colonic mucosa established the anti-inflammatory action of the propolis extract [65]. Moreover,* Baccharis dracunculifolia* DC (Asteraceae), a medicinal plant that is the main botanical source of Brazilian green propolis, also demonstrated a positive action in attenuating the colonic damage induced by TNBS in rats [66].

Dextran sodium sulfate (DSS) is used as the principal chemical agent for induction of intestinal inflammation in experimental animals, which has the ability to disrupt the integrity of the mucosal barrier. The main macroscopic marks include loss of weight, diarrhea, and rectal bleeding, while ulcerations and granulocyte infiltrations are the basic microscopic findings [63, 67]. In addition to the ability to induce UC, DSS is also used as a chemical agent to cause colon tumorigenesis, after a prior administration of carcinogenic initiators [68, 69]. Thus, Doi et al. evaluated the effects of ethanolic and water-extracts produced from Brazilian green propolis in the model of inflammation-associated rat colon carcinogenesis (1,2-dimethylhydrazine plus DSS treatment). The authors showed that the ethanol based extract exerted its anticancer effects through the suppression of inflammatory factors, such as tumor necrosis factor (TNF-*α*) and iNOS [70].

A recently published study evaluated the effects of caffeic acid phenethyl ester (CAPE), one of the main compounds of propolis, in DDS-induced acute colitis in a mice model. CAPE-treated group exhibited a protection in the epithelial barrier from disruption accompanied by and a decrease in MPO activity and proinflammatory cytokines levels [71]. In addition, CAPE effects were also explored in a previous study using the model of peptidoglycan-polysaccharide- (PG-PS-) induced colitis in rats [72]. PG-PS causes chronic inflammation, granulomas, crypt abscesses, and fibrosis, being also described as one of the only models that closely resemble Crohn's disease [73]. CAPE was able to attenuate the PGPS-induced colitis through its ability to inhibit the nuclear factor-*κ*B (NF-*κ*B) pathway, by reducing the production of proinflammatory cytokines and by induction of apoptosis in macrophages [72]. Notably, several authors have shown that NF-*κ*B was upregulated in macrophages and epithelial cells of patients with inflammatory bowel disease [74–76]. Furthermore, other studies have also shown the ability of CAPES as an inhibitor of NF-*κ*B activation [77–80]. Similarly, another compound found in propolis, the flavonoid quercetin, also demonstrated potential in inhibiting the NF-*κ*B pathway in an experimental model of DSS induced rat colitis [81]. In addition, quercetin has been the subject of several studies about its property in attenuating colitic damage in different experimental models, such as acetic acid-induced UC in mice [82, 83] and on TNBS-induced colitis in rats [84, 85].

It is well established that flavonoids are the main active constituents of propolis [86, 87]. This is in addition to those that have, already, been mentioned above (CAPE and quercetin). For this reason, the potential role of flavonoids found in propolis against UC has also been investigated. Of this class of compounds, the studies developed with kaempferol, luteolin, and naringenin stood out. Park et al. [88] showed that the treatment with kaempferol was effective against the damage induced by DSS in the colonic mucosa of mice, an effect related to its anti-inflammatory properties. Luteolin was able to ameliorate DSS-induced UC, which was verified in different experimental trials [89, 90]. Finally, naringenin showed a protective effect against DSS-induced UC in mice [91, 92] and in an acetic acid model of colitis in rats [93]. On the other hand, although propolis and its main components have shown promising results in the treatment of experimental UC (mainly due to their antioxidative and anti-inflammatory properties), as verified by the several publications described here, these studies do not translate to human application, remaining to be explored its efficacy and safety in clinical trials.

## 5. Propolis and Its Potential to Treat Gastrointestinal Cancers

When it comes to the therapeutic effects of honey and bee products on various types of cancers, several outstanding original scientific works on propolis research can be highlighted. Apoptosis is one of the most important homeostatic features of a biological system that has a therapeutic critical role against cancer. Apoptosis is mainly mediated via caspase-independent and caspase-dependent pathways which can be stimulated through extrinsic signals (TNF family of cytokine receptors) and intrinsic signals (cytochrome c from mitochondria) [94]. Most of studies on herbal medicines and natural products have been conducted to find out bioactive components that possess significant therapeutic effects against different types of cancer and to assess the anticancerous effects of propolis and its extracts, including either its ethanolic or its aqueous extracts.

Among the various ingredients of propolis, caffeic acid phenethyl ester (CAPE) and artepillin C are two that are well-studied components showing anticancerous effects, which impose their bioactivity through apoptotic pathway. Moreover, essential oils of propolis are able to suppress human tumors through the reduction on the cell proliferation. Essential oils extracted from Xinjiang propolis were able to cause cell cycle arrest and apoptosis induction in HTC-116 (a human colorectal cancer cell line) [95].

CAPE, commonly present in propolis, has various biological activities including cytostatic and cytotoxic properties [96]. Cytotoxic effects of CAPE have been reported against oral squamous cell carcinoma and oral epidermoid carcinoma-Meng 1 [97]. These effects in addition to DNA degradation are attributed to apoptosis and altered redox state [98]. The authors showed that, while there is no cell cycle arrest against normal human oral fibroblast, treatments with 25 *μ*M and 50 *μ*M of CAPE for 24 h cause arrest at G2/M phase and sub-G0/G1 peak, respectively, in OEC-M1 cells which is an oral squamous cell carcinoma cell line. The apoptotic effect of CAPE is also associated with a selective scavenging ability for hydrogen peroxide as shown in human leukemic HL-60 cell line study [99]. CAPE is a strong suppressor of TNF activating ability for NF*κ*B [100]. In fact, CAPE was able to suppress the activation of NF-*κ*B, which is referred to as “a Critical Link between Inflammation and Cancer” [101]. Further studies showed that CAPE has cytotoxic effects against human leukemia [102], oral submucous fibroblast, neck metastasis of gingiva carcinoma, and tongue squamous cell carcinoma cells [103]. On the other hand, the inhibition of NF-*κ*B implies apoptosis through Fas activation [104]. In BxPC-3 cells, a pancreas adenocarcinoma cell line, CAPE, was able to reduce mitochondrial transmembrane activity which leads to apoptosis through caspase activity of caspase-3/caspase-7 [105]. The therapeutic effect of CAPE against cholangiocarcinoma in extrahepatic biliary cancer cell line, human intrahepatic bile duct and human extrahepatic bile duct, intrahepatic bile ducts, and nonmalignant cholangiocyte cell line H69 showed that CAPE is able to inhibit NF*κ*B and induces apoptosis [106].

CAPE is also capable of scavenging free radical through 5-lipoxygenase inhibition [107] and suppressing lipid peroxidation [108]. Inhibition of NF*κ*B suppresses the level of inducible nitric oxide synthase and reduces its catalytic activity [109]. A study on CT26 colon adenocarcinoma showed that CAPE possesses angiogenesis effect that leads to the suppression of invasiveness on tumor cell and metastasis in murine [110]. Similarly, CAPE treatment of SKHep1 human hepatocellular carcinoma cells restricts invasion [111] and enhances glucose metabolism through adenosine monophosphate- (AMP-) activated protein kinase (AMPK) in skeletal muscle cells [112]. Self-renewal of breast cancer stem cells, isolated from MDA-231 cells which is a human triple-negative breast cancer model, demonstrated a dose-dependent inhibition by CAPE through the downregulation of the expression of CD44 (a marker of cancer-initiating cells in some malignancies [113]). The majority of these cells showed cell cycle arrest at G0/G1 level of the cell cycle [114].

In addition to CAPE, other ingredients of propolis such as artepillin C, galangin, kaempferol, and quercetin showed antiangiogenesis properties [115]. Artepillin C, found in Brazilian propolis, was able to suppress the formation of membranous lipid peroxidation and 8-hydroxydeoxyguanosine [116]. In colorectal cancer, CAPE was able to suppress *β*-catenin/Tcell factor signalling, an important malignancy marker [117], and to affect crypt foci and colorectal tumor in rats [118]. Moreover, in an* in vitro* study on colon cancer cell lines showed a suppressive effect of artepillin C on Cip1/p21 protein, a quiescence state of G0/G1 phase arrest, which in turn is a conquest of cytostatic state in colon cancer [119]. Propolis also contains prenylflavanone compounds such as propolin G, which showed some therapeutic effects against glioma and glioblastoma, brain cancer cell lines through caspases-dependent pathway of apoptosis, and mitochondria pathways [120].

Galangin is a flavonoid that can be found in propolis and has antigenotoxic properties which makes propolis a valuable bioactive agent against cancerous proliferation through mechanisms involving NF*κ*B, B-cell lymphoma-extra large [bcl-X(L)], and COX-2 (for a review see [121]). For instance, in human colon cancer cells (HCT-15 and HT-29), galangin is able to induce apoptosis and DNA condensation [122] and enhances “adenomatous polyposis coli gene product (APC)/Axin/glycogen synthase kinase-3 beta (GSK-3ß-) independent proteasomal degradation of ß-catenin” in adenomatous polyposis coli cancer cells and inhibits their proliferation [123]. Studies showed that galangin has suppressive effect against angiogenesis of ovarian cancer cells [124] and activates p38 MAPK and induces apoptosis through mitochondrial pathway in melanoma cells (B16F10) [125]. DNA fragmentation induced by galangin is another antiproliferative property of galangin as seen in HL-60 cells of a promyelocytic cell line [126].

Kaempferol is another flavonoid ingredient of propolis inducing apoptosis through activation of TNF-related apoptosis-inducing ligand (TRAIL) in SW480 cells, a human colon cancer [127], and inhibiting ribosomal protein S6 kinase (RSK2) and mitogen- and stress-activated kinase (MSK1), main regulators in tumor promoter induced cell transformation [128]. Moreover, this ingredient is able to suppress Src kinase activity and inhibits COX-2, through which kaempferol showed an effective preventive property against skin cancer [129]. Treatment with kaempferol causes a downregulation of proliferation in human prostate cancer through suppression of proliferating-cell nuclear antigen (PCNA) and vascular cell adhesion molecule-1 (VCAM-1) [130]. Similar to the other flavonoids ingredients, quercetin exhibits anticancerous properties through stimulation of apoptotic pathways. For instance, 25 *μ*M and 50 *μ*M of quercetin suppress the proliferation of prostate cancer cell lines such as PC-3 and DU-145 and stimulate tumor suppressor genes [131].

Therapeutic properties of propolis seem to vary according to their geographical location. For instance, Chinese and Korean propolis suppress interleukin- (IL-) 6 [132], which is a critical mediator of solid malignancies [133]. In mice, ethanolic extract of Brazilian propolis regulates the level of Toll-like receptor- (TLR-) 4 [134], promoting gastric cancer through production of mitochondrial reactive oxygen species (ROS) [135], and the level of IL-4 [136], promoting tumor growth and invasion [137]. The extract also showed cytotoxic in HEp-2, human laryngeal epidermoid carcinoma [138]. Methanol extract of red propolis showed significant cytotoxicity against human pancreatic PANC-1 cancer cell line [139]. Korean propolis contains ethanol-soluble ingredients that inhibit NF*κ*B [140] which is a potential anticancerous target [141]. Aqueous extracts of propolis inhibited proliferation of various cell lines such as McCoy, HeLa, SP2/0, HEp-2, and BHK21 [142]. Ethanolic extract of propolis contained more bioactive compounds than the water ones. Ethanolic extract of propolis increases TRAIL mediated apoptosis in malignant cells of human cervical cancer HeLa cell line [143], prostate cancer cells [144], and some human colon carcinoma cells such as CaCo2, HCT116, HT29, and SW480 [145]. Ethanolic extract of Polish to possesses chemopreventive effects against prostate cancer cells through apoptosis, which is activated by TRAIL receptor 2 [146].

## 6. Antiulcer Activity of Propolis

Gastric ulcer is defined as an injury to the gastric mucosa, which occurs due an imbalance between the luminal challenge exerted by the highly acidic and proteolytic properties of gastric juices and the ability of the mucosa to resist them [147]. This disease affects 10% of the world population, but its etiology is not completely understood [148]. There are various noxious agents to the stomach resulting in mucosal ulceration, such as* Helicobacter pylori* infections, prolonged ingestion of nonsteroidal anti-inflammatory drugs (NAIDs), alcoholic drinks, psychological stress, and cigarette smoking. On the other hand, the stomach protects itself through many defense mechanisms, mainly adequate blood flow and bicarbonate and mucus secretions [149].

The treatment of gastric ulcers is based on using antisecretory drugs, including type-2 histamine receptor antagonists (H2-RAs) and proton pump inhibitors (PPIs) [150], as well as antibiotics used to treat the* H. pylori* infections [151]. However, these therapeutic agents are typically associated with numerous adverse side effects, such as hypersensitivity, vitamin B12 and iron deficiency, arrhythmia, increased susceptibility to pneumonia, impotence, gynecomastia, bone fractures, hematopoietic changes, hypergastrinemia, and gastric cancer. In this context, natural products are considered as attractive sources for new antiulcer treatments. Among them, propolis has been used in folk medicine to treat gastric ulcer and this has boosted research in order to investigate and validate its use as an antiulcer agent as discussed below.

Investigations about the gastroprotective effects of ethanolic extract of propolis against ethanol-induced gastric ulcer in rats revealed that the administration of the extract prevented the occurrence of gastric ulcerations in a dose-dependent manner. Furthermore, propolis extract reduced the lipid peroxidation, based on both* in vivo* and* in vitro* experiments, and levels and scavenged the superoxide anion. So, the authors concluded that the gastric protective mechanism of ethanol propolis extract was due, at least in part, to its ability to protect the gastric mucosa from oxidative stress [152]. In another study, El-Ghazaly et al. [153] investigated the gastroprotective effect of an aqueous propolis extract which was evaluated using indomethacin-induced gastric ulcers in rats exposed or nonexposed to gamma radiation. The results from this study confirmed that the pretreatment with the aqueous propolis extract to the irradiated or nonirradiated rats protected against gastric ulceration. Moreover, the extract increased the mucosal prostaglandin E2 (PGE2) levels and decreased the TNF-*α* and IL-1*β* amount in the plasma. Interestingly, the gastric acid antisecretory effect of the aqueous propolis extract was described by those authors and the beneficial effects measured were associated with a reduction in acid output and peptic acid activity, associated with increased mucin secretion. Given that the therapeutic properties of propolis may vary according to the geographical collection region, it is important to emphasize that the extract used by El-Ghazaly and collaborators [153] was obtained using raw propolis from many different countries and standardized in 13% of propolis containing not less than 0.05% organic aromatic acids, calculated as the total of caffeic acid, ferulic acid, and cinnamic acid, beyond traces of different flavonoids.

The propolis produced in the Southeastern region of Brazil is known as green propolis because of its color. The plant* Baccharis dracunculifolia* DC (Asteraceae) is the primary source for it, a common species found in the Brazilian Cerrado. Due to similarities among chemical constituents of green propolis with those present in* B. dracunculifolia*, this plant was identified as being the principal source of green propolis. The antiulcer activity of green propolis hydroalcoholic crude extract was evaluated by de Barros et al. [154] using models of acute gastric lesions induced by ethanol, indomethacin, or stress in rats. In this study, the green propolis extract (500 mg/kg, p.o.) reduced the indomethacin-induced gastric ulcers. Moreover, in the stress-induced ulcer a significant reduction in ulcer area in animals treated with green propolis extract (250 and 500 mg/kg) was observed. Regarding the antisecretory capability of green propolis extract, the authors described a reduction in the gastric juice volume, as well as in the total acidity after extract (250 and 500 mg/kg) administration in pylorus ligated rats. Therefore, in accordance with the findings on propolis from other countries, these data place the Brazilian green propolis as a promising antiulcer agent. Barros et al. [155] also described the gastroprotective properties of the main phenolic acids found in Brazilian green propolis. Similarly to the results obtained by Barros et al. [155], the oral treatment with caffeic, ferulic, and p-coumaric and cinnamic acids at doses of 50 and 250 mg/kg diminished the total area of the lesion induced by different harmful agents. In addition, the effects of these substances on gastric acid secretions were measured and the results revealed that the phenolic acids tested, except for p-coumaric, reduced the gastric acid secretion in rats at a dose of 50 mg/kg.

Up to this point, the reviewed studies have evaluated the antiulcer protective effects of propolis preparations. Such findings do not necessarily mean that they has a healing capacity against gastric ulcers [156]. In view of this, chronic gastric ulcers induced by acetic acid instillation at gastric serosa have been a widely used model for the evaluation of the gastric healing potential of natural products or herbal medicines. Indeed, Belostotskiǐ and collaborators [157] described the healing gastric effects after administration of honey, royal jelly, and propolis in rats exposed to acetic acid into the gastric serosa. Based on the above, it would be prudent to recommend conducting further studies to strengthen the the healing potential of propolis based preparations or its constituents against gastric ulcers. This is in addition to having a more comprehensive understanding of its antiulcer activities and a better understanding of the underlying mechanism(s) of action.

Despite many studies about the antiulcer potential of propolis, mainly its gastroprotective action, little is known regarding its activity against* H. pylori*. In this field, Villanueva et al. [158] evaluated the inhibitory activity of 22 propolis extracts obtained from nine of the 11 beekeeping Chilean regions on 10 strains of* H. pylori* isolated from the gastric mucosa. Interestingly, 100% of the tested extracts inhibited the* H. pylori* growth, but those authors also pointed out that the need for additional microbiological studies before a potential clinical trial of these natural products is warranted.

## 7. Conclusions

In conclusion, this review included a summary of the data published by many researchers related to the protective and/or treatment role that propolis and/or its active ingredients play against gastrointestinal associated disorders that affect humans. The focus was on the following: oral mucositis, ulcerative colitis, gastrointestinal cancers, and gastric ulcers. Analysis of the published work indicated that the efficacy of propolis in the treatment of gastrointestinal disorders could be attributed to its antioxidants and anti-inflammatory properties. The underling mechanism of action is mediated through the inhibition of some transcriptional factors and related proteins. Several experimental studies showed the beneficial effects of propolis and its related compounds in the treatment of gastrointestinal diseases. However, only few clinical trials have been developed to prove their effectiveness and safety against human ulcers and other involved pathologies. Future studies should focus on the potential role of propolis and its related ingredients either alone or as a complementary therapy to ongoing conventional therapy against gastrointestinal diseases in humans.
